# Neonatal blood transfusion practices in India: a nationwide survey of clinicians

**DOI:** 10.3389/fped.2025.1692244

**Published:** 2025-11-18

**Authors:** Karthik S. Kolkur, Suprabha K. Patnaik, Nandini Malshe, Vikrant Deshmukh, Shalini Tripathi, Nandini Nagar, Sreevidya Sreekantha, Pradeep Suryawanshi

**Affiliations:** 1 Department of Neonatology, Bharati Vidyapeeth Deemed to be University Medical College, Pune, Maharashtra, India; 2 Department of Pediatrics, KGMU, Lucknow, Uttar Pradesh, India; 3 Department of Neonatology and Pediatrics, Cloudnine Hospital, Bengaluru, India; 4 Department of Neonatology, Motherhood Women and Children’s Hospital, Bengaluru, India

**Keywords:** blood transfusion, India, neonate, NICU, survey

## Abstract

**Background:**

Blood transfusion, a vital procedure in neonatal intensive care units (NICUs), also poses risks such as necrotizing enterocolitis, intraventricular hemorrhage, and death. We conducted a nationwide survey to evaluate current neonatal transfusion practices among clinicians in India.

**Methods:**

A cross-sectional survey with a structured 23-item questionnaire was conducted online using Google Forms during February 2024. The questionnaire covered key elements of transfusion practices, including threshold, dosing, and duration of blood product administration. Five hundred forty clinicians working in NICUs across India were invited to participate in the survey.

**Results:**

Responses were received from 368 clinicians, most of whom practice in Level 3 Neonatal Intensive Care Units. Approximately 67% reported adherence to the guidelines established by the National Neonatal Forum of India 2020. Packed red blood cells were predominantly transfused at a volume of 15 mL/kg (79%) over a duration of four hours (69%), with a hemoglobin threshold of 7.5 g/dL (48%) employed after two weeks of life in stable preterm neonates younger than 32 weeks. The majority of practitioners (59%) did not utilize diuretics, and half (50%) withheld feeds during red blood cell transfusions. Platelet transfusions were most frequently administered at 10 mL/kg (51%) over a period of 0.5 h (60%), with a threshold platelet count of 50,000/µL in cases of bleeding (60%) or 25,000/µL in the absence of bleeding (58%). Fresh frozen plasma was used in neonates presenting with coagulopathy and bleeding (73%) and also in cases without bleeding (25%), most commonly at 10 mL/kg (47%) administered over 1 h (43%).

**Conclusion:**

Transfusion practices varied across Indian NICUs despite adherence to NNF guidelines and generally adopting a restrictive approach. The standardization of protocols and enhancement of compliance could potentially improve clinical outcomes and diminish complications associated with transfusion.

## Introduction

Blood transfusion is a common intervention used in the neonatal intensive care unit (NICU) for premature and sick newborns. During the transition from a fetus to a neonate, several physiologic changes occur, including changes in blood volume and other hematologic parameters (1). In extremely low birth weight infants, maintaining a higher hemoglobin level results in more infants receiving transfusions, although there is little evidence that this is beneficial (2). Anemia may result in impaired oxygen supply to the brain and prematurity-related brain injury, especially in the setting of apnea and intermittent hypoxemia or circulatory insufficiency during a period of rapid brain growth and development. However, red blood cell (RBC) transfusions can have complications, especially in preterm infants. They have been associated with intraventricular hemorrhage, necrotizing enterocolitis (NEC), bronchopulmonary dysplasia, retinopathy of prematurity, and increased mortality (3).Transfusions in preterm neonates may be harmful due to pro-inflammatory and immunosuppressive effects or, as recently proposed, from repeated adult red blood cell (RBC) transfusions that replace fetal hemoglobin (HbF) with adult hemoglobin, increasing oxidative stress. This oxidative stress promotes band 3 oxidation and neoantigen formation, facilitating phagocytic recognition (4). Supporting this, Pellegrino et al. showed that adult donor RBCs deliver more oxygen to preterm neonatal brains than cord blood (5).

Many clinicians use a specific hematocrit or hemoglobin level to assess the need for RBC transfusion, which varies based on the degree of prematurity, the postnatal age, and the clinical state of the infant, including oxygen requirement and need for ventilatory support (6). Two large randomized clinical trials (ETTNO and TOP) have been published since 2019 regarding transfusion threshold and neurodevelopmental outcome in neonates (7). In extremely low birth weight infants, a higher hemoglobin threshold for red blood cell transfusion did not improve survival without neurodevelopmental impairment at 22–26 months of age, corrected for prematurity (TOP trial) (8). Among infants with birthweight <1,000 g, a liberal blood transfusions strategy compared to a restrictive approach did not reduce the risk of death or disability at 24 months of corrected age (9).

In India, large variation in platelet transfusion practice has been demonstrated, regarding the thresholds used, the volume administered, and the transfusion rate (10). In the PLANET 2 trial, preterm infants with low platelet count who received platelet transfusions at a higher threshold were found to have an increased risk of death or major bleeding compared to those who were transfused at a lower threshold (11).

Over the past 20 years, several recommendations for blood transfusion in newborns have been published (11–13). Despite the availability of neonatal transfusion guidelines, there is a lack of Indian data on transfusion practices like threshold, dosing, and duration of different blood products used in NICUs across India. Therefore, this study aimed to evaluate current neonatal transfusion policies and practices in Indian neonatal critical care units.

## Material and methods

The study started in February 2024 after getting approval from the institutional ethical committee. An online survey with 23 multiple-choice questions about blood transfusion practices and policies was created using Google Forms and tested for content validity by five practicing neonatologists. After finalizing, clinicians across India (*n* = 540), listed in the directories of the National Neonatology Forum (NNF) of India and the Neonatology Chapter of the Indian Academy of Pediatrics, were contacted by phone, text, and email to participate. Ten of the twenty-three questions were examined to see if public and private hospitals followed the NNF recommendations for transfusion practices. Adherence to the NNF guidelines was evaluated using a 10-item questionnaire. Each item was scored as 1 if the NICU followed the respective guideline and 0 if it did not. The total adherence score was calculated by summing the responses across all items, with higher scores indicating better compliance. Participants implicitly consented to the survey. All information received was kept confidential. After a month of weekly reminders, the survey was closed. No incentives were offered. The responses are summarized in Tables 1–4. The questionnaire is provided in the Supplementary File.

**Table 1:** Characteristics of survey participants (*n* = 368).

Characteristic	*n*	%
Designation		
Neonatologist	135	36.6
General pediatrician	130	35.3
Senior pediatrics resident	103	28.1
Type of organization housing the neonatal intensive care unit		
Private (non-government-run) hospital	211	57.4
Public (government-run) medical college	74	20.1
Private medical college	49	13.3
Public hospital	34	9.2
NICU category as per National Neonatology Forum of India		
Level 3A	139	37.8
Level 2	81	22.0
Level 3B	80	21.7
Level 3C	46	12.5
Level 1	22	6.0
Follow guidelines for neonatal blood transfusion		
Yes	341	92.7
No	27	7.3
Guidelines followed (*N* = 341)		
National Neonatology Forum of India	246	72.1
American Association of Pediatrics	50	14.7
National Institute for Healthcare and Excellence, UK	37	10.9
Other	8	2.3

**Table 2:** Red blood cell (RBC) transfusion practices of survey participants in their neonatal intensive care units (*n* = 368).

Characteristic	*n*	%
Blood product transfused for anemia		
Packed RBC (PRBC)	362	98.4
Whole blood	6	1.6
Hemoglobin threshold for PRBC transfusion in 1st week of life in ventilated preterm neonates of age < 32 weeks		
10 g/dL	144	39.1
12 g/dL	142	38.6
11 g/dL	45	12.2
9 g/dL	37	10.1
Hemoglobin threshold for PRBC transfusion in 1st week of life in preterm neonates of age < 32 weeks on non-invasive respiratory support		
10 g/dL	202	54.9
9 g/dL	101	27.5
11 g/dL	34	9.2
12 g/dL	31	8.4
Hemoglobin threshold for PRBC transfusion after 2nd week of life in stable preterm neonates of age < 32 weeks		
7.5 g/dL	178	48.3
8 g/dL	103	28.0
10 g/dL	54	14.7
9 g/dL	33	9.0
PRBC transfusion dosing		
15 mL/kg	290	78.8
20 mL/kg	55	14.9
5 mL/kg	23	6.3
PRBC transfusion duration		
4 h	254	69.0
3 h	75	20.4
5 h	22	6.0
6 h	17	4.6
Use of diuretics with blood transfusion		
No	218	59.2
Yes	150	40.8
Enteral feeding during RBC transfusion		
Yes	185	50.3
No	183	49.7

**Table 3:** Platelet transfusion practices of survey participants in their neonatal intensive care units (*n* = 368).

Characteristic	*n*	%
Platelet count threshold for preterm neonates with clinical bleeding		
50,000/µL	222	60.3
100,000/µL	89	24.2
25,000/µL	55	14.9
10,000/µL	2	0.6
Platelet count threshold for preterm neonates without clinical bleeding		
25,000/µL	212	57.6
10,000/µL	107	29.1
50,000/µL	41	11.1
100,000/µL	8	2.2
Platelet count threshold for transfusion prior to lumbar puncture		
50,000/µL	172	46.7
25,000/µL	93	25.3
30,000/µL	61	16.6
10,000/µL	42	11.4
Platelet transfusion duration		
0.5 h	220	59.8
1 h	139	37.8
2 h	9	2.4
Platelet transfusion dosing		
10 mL/kg	187	50.8
15 mL/kg	146	39.7
20 mL/kg	33	9.0
>20 mL/kg	2	0.5
Routine platelet transfusion for closure of patent ductus arteriosus in thrombocytopenic preterm neonates		
No	346	94.0
Yes	22	6.0

**Table 4:** Fresh frozen plasma (FFP) transfusion practices of survey participants in their neonatal intensive care units (*n* = 368).

Characteristic	*n*	%
Indication of FFP transfusion		
Deranged coagulation with bleeding	269	73.1
Deranged coagulation without bleeding	95	25.8
Hypotension	4	1.1
FFP transfusion dosing		
10 mL/kg	173	47.0
15 mL/kg	161	43.8
20 mL/kg	34	9.2
FFP transfusion duration		
1 h	159	43.2
0.5 h	113	30.7
2 h	96	26.1

## Results

### Characteristics of survey respondents

**Figure 1:**
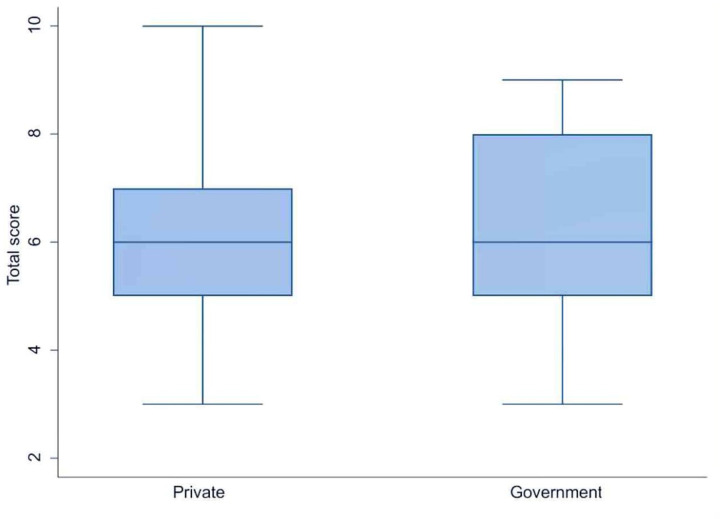
Comparision of adherence to transfusion practices in private and public hospitals in India.

Of the 540 clinicians invited to participate, 368 (68.1%) responded to the online survey. They came from 260 institutions and hospitals across 65 cities in 25 states of India. Table 1 shows the characteristics of the survey respondents. Most respondents were neonatologists (37%) or general pediatricians (35%). The importance of private healthcare in newborn services was reflected in institutional statistics showing that private (non-government) hospitals housed the majority of NICUs (57%), where the respondents worked, followed by government (public) medical colleges (20%) and private medical colleges (13%).The mean adherence score to the NNF guidelines was 6.27 ± 1.44 among clinicians working in private hospital (*n* = 263) and 6.31 ± 1.51 among those in government hospital(*n* = 105), with no statistically significant difference between the two groups (*p* = 0.81) (Figure 1). Seventy-two percent of the NICUs were classified as NNF India category Level 3A or higher, indicating a strong focus on intensive and specialized neonatal care according to NNF accreditation standards. The survey respondents used various transfusion protocols, with 90.6% following guidelines from either NNF India (67%), the American Academy of Pediatrics (13.6%), or the National Institute for Healthcare and Excellence (NICE) of the UK (10%).

### RBC transfusion practices

Survey responses on RBC transfusion practice are summarized in Table 2. Packed Red blood cell (PRBC) was the blood product of choice of 98% of respondents for the management of anemia. As suggested in NNF India guidelines, PRBC transfusion at a hemoglobin threshold of 7.5 g/dL after the second week of life in stable preterm infants of age < 32 weeks was performed by 48% of respondents. For preterm infants of age < 32 weeks in first week of life, hemoglobin threshold that was used was 10 g/dL (39%) or 12 g/dL (39%) for those on ventilator, while it was 10 g/dL (54.9%) for those on non-invasive support. There is limited evidence to determine the ideal PRBC transfusion dosing for newborns, particularly concerning long-term outcomes. In our survey, PRBCs were transfused most frequently (79%) at 15 mL/kg, as recommended in the NNF 2020 guidelines, with 15% and 6% of respondents respectively choosing dosings of 20 mL/kg or 5 mL/kg. PRBC was typically transfused over four hours in most cases (69%). Diuretics were used by 41% of respondents during transfusion, whereas 50% of respondents delayed enteral feeding during RBC transfusion, in line with NNF India guidelines.

### Platelet transfusion practices

About 60% of respondents transfused platelets at a threshold platelet count of 50,000/µL in preterm newborns with clinical bleeding (Table 3). In the absence of such bleeding, a threshold of 25,000/µL was used by a majority (58%), as recommended in the guidelines of NNF India as well as British Society of Haematology (BSH) (12). Additionally, as per BSH guidelines, a threshold of 50,000/µL was used by 47% of respondents for platelet transfusions prior to lumbar puncture. Most (94%) respondents did not routinely transfuse platelets in thrombocytopenic preterm newborns for closure of patent ductus arteriosus (PDA), in accordance with NNF India guidelines. Platelet transfusions were administered at a dosing of 10 mL/kg (51%), 15 mL/kg (40%), or 20 mL/kg (9%). Sixty percent of respondents administered transfusions over 0.5 h and 40% over one hour.

### Fresh frozen plasma (FFP) transfusion practices

For FFP transfusion, 73% of respondents followed NNF India guidelines by transfusing in neonates with deranged coagulation and active bleeding, while 26% transfused in cases of deranged coagulation without bleeding (Table 4). FFP was transfused at a dose of 10 mL/kg (47%), 15 mL/kg (44%), or 20 mL/kg (9%), over a period of 1 h (43%), 0.5 h (31%), or 2 h (26%).

## Discussion

Transfusion of blood products is a common intervention used in the NICU to treat premature and ill newborns. RBC transfusions are often recommended in the early weeks of infancy to treat anemia, which improves oxygen delivery and cardio-respiratory function status. Conversely, the adverse effects of RBC transfusion are increasingly recognized (13). Despite the growth in the number of Neonatal Intensive Care Units (NICUs), the specific operational practices of these facilities remain inadequate. In the absence of a system of mandatory registration and assessment by authorities, one has to rely on periodic surveys for an overview of the current situation regarding transfusion procedures and policies in Indian NICUs (14–16). Our study provides an insight into different neonatal blood transfusion practices among clinicians in India.

A small percentage of respondents reported continued use of whole blood for neonatal anemia even though it has largely been replaced by packed red blood cells (PRBCs). This may indicate difficulty in blood component separation or logistical issues in preparing PRBCs, particularly in smaller centers. In our survey, 40% of clinicians reported using diuretics during transfusions (Table 2), despite limited evidence of benefit. Randomized trials, including Balegar et al., show minimal clinical advantage, suggesting this practice persists due to concern about fluid overload or institutional policies rather than evidence-based guidance (17).

In our cohort, 50% of clinicians withheld feeds during PRBC transfusions, reflecting moderate guideline adherence, while evidence indicates uncertain NEC protection and possible harms such as disrupted nutrition and additional interventions (13, 14, 18, 19).

Beyond variations in diuretic use and enteral feeding practices, survey responses also revealed differences in transfusion thresholds, reflecting ongoing uncertainty and heterogeneity in clinical decision-making despite established guidelines. In stable preterm neonates <32 weeks, 48% of clinicians reported using a hemoglobin threshold of 7.5 g/dL for transfusion after the second week of life. Evidence indicates that restrictive thresholds (7–8 g/dL) do not increase mortality or adverse outcomes compared to liberal thresholds (9–10 g/dL), and higher thresholds increase transfusion frequency without improving long-term neurodevelopmental outcomes (9, 20). These findings highlight the need to individualize transfusion decisions based on clinical condition, oxygen requirements, and hemodynamic stability.

For preterm infants, maximum number of transfusions occurred during first and second weeks of life (21). For PRBC transfusion in the first week of life in ventilated preterm neonates of age < 32 weeks, the majority of practitioners in our survey used a hemoglobin threshold of 10 g/dL, in contrast to 12 g/dL suggested in NNF India guidelines (19). However, a majority used a threshold of 10 g/dL in case of neonates on non-invasive respiratory support, in accordance with the NNF guidelines. Similar results were found by Bell et al. in their investigation (22).

In VLBW infants, most RBC transfusions are top-ups to replace phlebotomy losses, and volumes >20 mL/kg in non-bleeding neonates may increase the risk of fluid overload (21). Conservative transfusion volumes of 15 mL/kg are generally appropriate, as studies show that larger volumes (15–20 mL/kg) can reduce the need for multiple transfusions and donor exposure without increasing adverse events or affecting pulmonary function (23–25). These findings support individualized, conservative transfusion strategies that balance efficacy with safety in this vulnerable population.

Alongside variations in RBC transfusion practices, our survey also revealed conservative platelet transfusion thresholds. with 58% of clinicians using 25,000/µL in non-bleeding neonates. This is lower than thresholds reported in previous studies (30,000–50,000/µL) (26–28), indicating closer adherence to NNF guidelines and a more conservative, evidence-informed approach. The BSH-recommended platelet threshold of 25,000/µL for prophylactic transfusions in non-bleeding preterm neonates is supported by the PLANET-2 trial and recent RCTs, which reported higher mortality and neurodevelopmental impairment at 50,000/µL (11, 29) In our survey, 89% of respondents used platelet thresholds of 25,000–50,000/µL, consistent with previous reports (30), although a majority (47%) used 50,000/µL prior to lumbar puncture, reflecting cautious practice in procedures with potential bleeding risk (31). Only 6% reported transfusing platelets for PDA closure, in line with evidence showing that higher platelet counts do not facilitate early PDA closure (32). Clinicians remain aware of transfusion-related risks, including acute lung injury and allergic reactions (33).

Following platelet transfusion practices, our survey assessed FFP use, which is particularly relevant in extremely low birth-weight neonates with coagulopathy or active bleeding (34). We observed that 73% of clinicians administered FFP for deranged coagulation with active bleeding, while 26% transfused in the absence of bleeding (Table 4), highlighting variability in practice and potential overuse. The lack of defined optimal dosing guidelines further underscores the need for evidence-based protocols to guide FFP transfusion and minimize unnecessary exposure in this vulnerable population.

To our knowledge this is the first cross-sectional study among practicing clinicians and neonatologists depicting transfusion practices and policies from various NICUs across India. The responses from clinicians nationwide provide broad geographic coverage. Although this is a comprehensive questionnaire-based study, most questions were answered by respondents, with an option for free-text input to share opinions on different practices beyond the provided alternatives. There are several limitations to our study. Firstly, its findings may not be a true reflection of practices across the country because of inadequate sampling. Secondly, some responses regarding transfusion policies were based on personal opinions rather than standardized national guidelines. Seventy-one percent of participants were affiliated with private hospitals or medical colleges. This bias can skew our findings as the practices in private institutions may differ from those in public ones, particularly in terms of resource availability, patient population, and staff training. The choices for transfusion thresholds, dosings, and rates in the survey questionnaire were not objectively validated, which may restrict the accuracy and practicality of the survey's findings. Furthermore, variables such as hospital infrastructure, staff experience, and financial status of patients that may have an impact on transfusion procedures were not taken into consideration in this study. The cross-sectional nature of the study limits the ability to establish temporal relationships or infer causality between the observed variables.

In conclusion, our study emphasizes the differences in neonatal blood transfusion practices across India, even though most practitioners report following NNF India guidelines. Its findings indicate that healthcare providers should regularly review and update their transfusion protocols based on the latest evidence and guidelines, while also ensuring compliance and conducting regular audits of their transfusion practices. The study further underscores the need for greater standardization of neonatal transfusion protocols to reduce transfusion-related complications and enhance neonatal outcomes.

## Data Availability

The original contributions presented in the study are included in the article/Supplementary Material, further inquiries can be directed to the corresponding author.
