# Supplementary material for: Neonatal blood transfusion practices in India: a nationwide survey of clinicians
Source: Front Pediatr. 2025 Nov 18;13:1692244. doi: 10.3389/fped.2025.1692244 (PMC12668949; doi:10.3389/fped.2025.1692244)
Supplement: Supplementary file 1 [file Datasheet1.pdf]

NEONATAL TRANSFUSION PRACTICES AND POLICIES IN INDIA-A QUESTIONNAIRE STUDY

Ko

In India, majority of the neonatal blood transfusions are often prescribed on expert clinical opinion rather than concrete documented guidelines. Transfusion guidelines in neonates varies greatly worldwide and between institutions. Our survey aims to analyze neonatal transfusion practices and policies in Indian NICU's.

We thank you for your participation.All information that is filled in by you will remain confidential.

* Indicates required question

1.

Mark only one oval.

- Option 1

2.

Mark only one oval.

- Option 1

Untitled title

3. 1. Name of the Institution/City/State *

4. 2. Name and designation of the person filling out this survey *

____

5. 3.What is the center/ institution where your NICU is located? *

Mark only one oval.

- Government Medical College
- Private Medical College
- Government Hospital
- Private Hospital

6. 4)which of the following category does your NICU belongs to as per NNF accredition norms? *

Mark only one oval.

- Level 1
- Level 2
- Level 3A
- Level 3B
- Level 3C

7. 5. Do you follow any guidelines for blood transfusion in neonate? *

Mark only one oval.

- Yes
- No

8. 6. If your answer to above question is yes, which guidelines do you follow in your unit? *

Mark only one oval.

- NNF2020
- NICE2015
- AAP
- Other: ______________________

9. 07. Do you routinely use diuretic along with blood transfusion in your unit? *

Mark only one oval.

- Yes
- No

10. 08. Do you withheld enteral feeds during red blood cell transfusion in your unit? *

Mark only one oval.

- Yes
- No

11. 09. which blood product will be given in your unit in case of anemia? *

Mark only one oval.

- PCV
- Whole blood

12. 10. What volume of pack red cell would you transfuse in your neonatal unit? *

Mark only one oval.

- 5ml/kg
- 15ml/kg
- 20ml/kg
- >20ml/kg

13. 11. Pack red blood cell transfusion is given over how much duration in your unit? *

Mark only one oval.

- 3hours
- 4hours
- 5hours
- 6hours

14. 12. what is the hemoglobin threshold for PRBC transfusion after 2nd week of life in stable preterm neonates <32weeks followed in your unit? *

Mark only one oval.

- 7.5g/dl
- 8g/dl
- 9g/dl
- 10g/dl

15. 13. What is the threshold level of platelets would you consider to transfuse before lumbar puncture in your unit? *

Mark only one oval.

- <10,000
- <25,000
- <30,000
- <50,000

16. 14. what is the hemoglobin threshold in first week of life for PRBC transfusion in ventilated preterm neonates <32 weeks followed in your unit? *

Mark only one oval.

- 9g/dl
- 10g/dl
- 11g/dl
- 12g/dl

17. 15. what is the hemoglobin threshold in first week of life for PRBC transfusion in preterm neonates <32 weeks on non invasive support? *

Mark only one oval.

- 9g/dl
- 10g/dl
- 11g/dl
- 12g/dl

18. 16) What is the threshold level of platelets would you consider to transfuse in preterm neonate with clinical bleeding? *

Mark only one oval.

- 10000
- 25000
- 50000
- 1,00,000

19. 17) What is the threshold level of platelets would you consider to transfuse in preterm neonate without clinical bleeding? *

Mark only one oval.

- 10,000
- 25,000
- 50,000
- 1,00,000

20. 18. Platelet transfusion is given over how much duration in your unit? *

Mark only one oval.

- 30 min
- 1 hour
- 2 hours

21. 19. what is the volume of platelets transfused in your unit ? *

Mark only one oval.

- 10ml/kg
- 15ml/kg
- 20ml/kg
- >20ml/kg

22. 20. Does your unit routinely transfuse platelets for PDA closure in thrombocytopenic preterm neonates with PDA? *

Mark only one oval.

- yes
- No

23. 21.What is indication of FFP transfusion in your unit? *

Mark only one oval.

- Deranged coagulopathy with active bleeding
- Deranged coagulopathy without bleeding
- Hypotension

24. 22. what is the volume of FFP transfused in your unit? *

Mark only one oval.

- 10ml/kg
- 15ml/kg
- 20ml/kg
- >20ml/kg

25. FFP transfusion is given over how much duration in your unit? *

Mark only one oval.

- ( ) 30min
- ( ) 1 hour
- ( ) 2 hours

This content is neither created nor endorsed by Google.

Google Forms
